# A New Face of Fibrin-Associated Large B-Cell Lymphoma: Epstein–Barr Virus-Positive Breast Implant-Associated Diffuse Large B-Cell Lymphoma

**DOI:** 10.3390/jcm12113614

**Published:** 2023-05-23

**Authors:** Jose Manuel Martin de Bustamante, Ana Mendoza, Samuel López-Muñoz, Eugenia García-Fernández, Pilar Gómez-Prieto, Victor Jiménez-Yuste

**Affiliations:** 1 Department of Hematology, IdiPaz, La Paz University Hospital, 28046 Madrid, Spain; anamendoz94@gmail.com (A.M.);; 2 Department of Pathology, La Paz University Hospital, 28046 Madrid, Spain; 3 Autónoma University, 28046 Madrid, Spain

**Keywords:** diffuse large B-cell lymphoma, Epstein-Barr virus, breast implants, fibrin-associated diffuse large B-cell lymphoma

## Abstract

Recently, there have been reports of what could be a new lymphoproliferative entity: breast implant-associated Epstein–Barr virus positive (EBV+) diffuse large B-cell lymphoma (EBV+ BIA-DLBCL). The new World Health Organization classification has categorized it as fibrin-associated large B-cell lymphomas (FA-LBCLs); therefore, it could be referred to as breast implant-associated fibrin-associated large B-cell lymphomas (BIA-FA-LBCLs). Although the association between breast implants and lymphomas has been known since the mid-1990s, it has been almost exclusively breast implant-associated anaplastic large cell lymphoma (BIA-ALCL). Here, we describe the first case of BIA-FA-LBCL at our center, with a literature review of the clinical features, diagnosis and treatment approach of this lymphoma. We also explore the differential diagnosis of BIA-FA-LBCL, highlighting the diagnostic challenges and the reasons that have led these lymphomas to being labeled as a new face of FA-LBCL.

## 1. Introduction

Breast lymphomas are a rare but known group of neoplasms that can be divided into non-breast-implant-associated lymphomas and breast-implant-associated (BIA) lymphomas. Non-breast-implant-associated lymphomas represent less than 0.5% of all breast malignancies and approximately 1% of all non-Hodgkin lymphomas [1]. They have predominately B-cell histology and require aggressive management. In contrast, BIA lymphomas are mostly of T-cell origin, with BIA anaplastic large cell lymphoma (BIA-ALCL) taking the spotlight. BIA-ALCL has been recognized as an entity for the first time by the World Health Organization (WHO) in their 2017 classification of Tumors of Hematopoietic and Lymphoid Tissues [2], and it has been ratified in the new WHO [3,4] and International Consensus Classification (ICC) [5] categorization of tumors. This lymphoma is normally detected at an early stage and has good progression free survival (PFS) and overall survival (OS) with surgery, only requiring chemotherapy in advanced stages [6,7,8]. Until recently, there were few reports of B-cell lymphomas related to breast implants, with no clear predominance of any subtype [1,9]. Since 2019, however, reports of diffuse large B-cell lymphoma (DLBCL) cases related to breast implants have clearly increased [9,10,11,12,13,14,15,16]. What stands out about these DLBCL cases is their positivity for Epstein–Barr virus (EBV) and their good prognosis with surgical management [10,11,12,13,14,15,16]. These findings differ from EBV+ diffuse large B-cell lymphoma (EBV+ DLBCL, also known as EBV+ DLBCL-not otherwise specified (NOS) in the ICC classification), which has an aggressive clinical presentation and poor prognosis [17,18,19,20]. Since the first reports of these BIA EBV+ DLBCL (EBV+ BIA-DLBCL) cases, it was theorized that they could be a new presentation of DLBCL associated with chronic inflammation (CI-DLBCL) or fibrin-associated LBCL (FA-LBCL) [1,11,12,13,16]. Although the most recent WHO classification has clearly labeled them as FA-LBCL [3], the ICC has not addressed this issue [5]. Despite the classification, the diagnosis can still be complicated, and clinicians and pathologists should be aware of this new face of FA-LBCL. We agree with the WHO classification; thus, we will now refer to these EBV+ BIA-DLBCLs as breast implant fibrin-associated large B-cell lymphomas (BIA-FA-LBCLs) during this review. Given that our goal is to focus on FA-LBCL arising in the context of a breast implant, and not all FA-LBCLs, the term EBV+ BIA-DLBCL could lead the reader to mistakenly believe that these lymphomas are a subtype of EBV+ DLBCL. Here we describe the first case of BIA-FA-LBCL at our center, its clinical presentation and how we approached it following the literature published until March 2023.

## 2. Case Report

A healthy, immunocompetent, 42-year-old woman had breast augmentation surgery with bilateral textured breast implants, performed for cosmetic reasons in 2014. Seven years later, she developed pain in both breasts, and she was diagnosed with bilateral capsular contracture, grade III. After 6 months of conservative management without improvement, in July 2021 she had surgical capsulectomy and implant replacement in both breasts. The capsules were sent for analysis to the pathology department; after their evaluation had been completed, she was referred to the hematology department.

**Figure 1:**
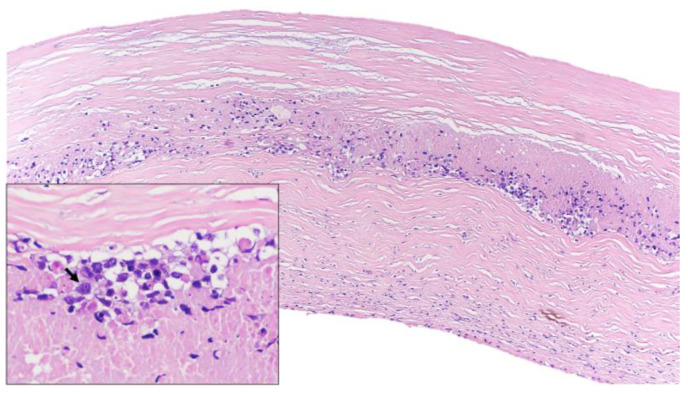
Histological features (hematoxylin and eosin). Lymphoid infiltrate inside the fibrotic capsule of the breast implant. At higher magnification, lymphoid cells were large and pleomorphic (arrow) and necrosis was identified.

**Figure 2:**
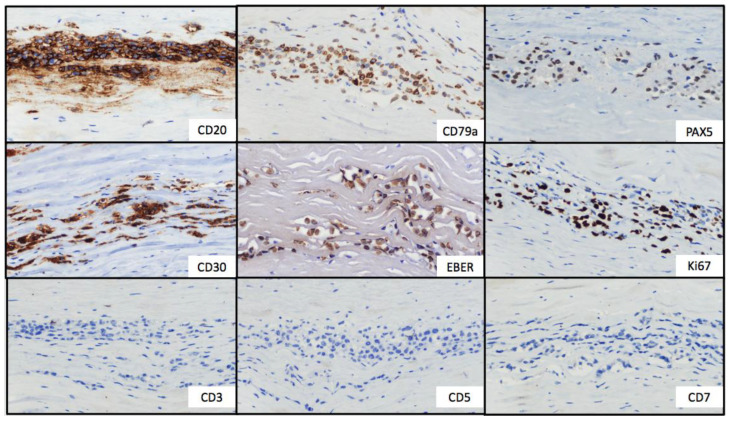
Immunohistochemical stains. Tumor cells showed strong and uniform staining of B-cell markers (CD20, CD79a and PAX5) and CD30. In situ hybridization for EBV-encoded small RNA showed positive labeling of the lymphoma cells. The Ki67 proliferation index was positive in approximately 50% of neoplastic cells. T-cell markers (CD3, CD5 and CD7) were negative.

Gross examination of the right capsule showed a smooth and pale surface, without interior fluid content. Histologic evaluation revealed a reactive inflammatory infiltrate associated with fibrin deposits. In contrast, the gross examination of the left capsule revealed rough, dark gray tissue. The histologic evaluation showed microscopic clusters of large, atypical and pleomorphic lymphocytes with prominent nucleoli located within the capsule, associated with areas of fibrin deposits and necrosis (Figure 1). No infiltration of adjacent tissues or mass formations were found. By immunohistochemistry, the atypical cells were positive for CD30, CD20, CD19, CD79a, PAX-5, BCL2 and MUM1; with no expression of CD3, CD4, CD5, CD8, CD10, Cyclin D1, CD13, ALK or TdT (Figure 2). Fluorescent in situ hybridization (FISH) analysis excluded BCL-2, BCL-6 and MYC rearrangements. The Ki-67 proliferation index was positive in approximately 50% of neoplastic cells. In situ hybridization for EBV-encoded small RNA (EBER) was positive; however, neither EBNA2 nor LMP1 were available in our laboratory for determining the latency pattern.

These findings led, at that time, to the diagnosis of EBV+ BIA-DLBCL (now BIA-FA-LBCL). Due to the unusual diagnosis of this type of lymphoma, which was not included in the WHO classification at the time [2], a review of the literature was necessary to confirm the diagnosis. The patient had normal blood counts, with normal levels of serum lactate dehydrogenase (LDH) and beta 2 microglobulin. The systemic examination was completed with a positron emission tomography scan that did not show any pathological uptake, ruling out the presence of disseminated disease.

Due to the possible indolent nature of this entity and based on the literature that had been published at that time, the multidisciplinary decision was to perform active surveillance after the surgical intervention. Prophylactic removal of the new implants was performed in September 2021, without signs of lymphoma in the histological analysis of both capsules. The patient is currently under follow-up, and after almost 2 years, she is healthy and with no evidence of recurrence.

## 3. Materials and Methods

A literature review was performed to retrieve the published cases of BIA-FA-LBCL from 1990 to March 2023. Given that all of them had been labeled as EBV+ BIA-DLBCL, the terms “breast implant-associated lymphoma”, “breast diffuse large B-cell lymphoma”, “Epstein-Barr virus diffuse large B-cell lymphoma” and various combinations of these terms and their abbreviations were used during the research. The consulted databases were PubMed, Web of Science and Embase.

A review of the clinical, histological and treatment characteristics of BIA-ALCL, EBV+ DLBCL, CI-DLBCL and FA-LBCL was also performed, to compare those lymphomas with our findings of the BIA-FA-LBCLs and to establish the differential diagnosis.

## 4. Results

Reports of BIA lymphomas began during the early 1990s [1,21]. Although the primary pathology described has been BIA-ALCL, recently, several cases of B-cell lymphomas related to breast implants have been published, predominantly with DLBCL histology [1]. In contrast to the extensive literature on BIA-ALCL, evidence for BIA-DLBCL is limited, with only a few cases published since 2014 [1]. Furthermore, EBV positivity has been reported in most cases, which suggests a significant association between EBV and these lymphomas [16].

In 2020, Mescam et al. published a case series of BIA-FA-LBCLs (as EBV+ BIA-DLBCLs) [11]; however, the actual first case of BIA-FA-LBCL had been reported by Goodwins et al. in 2019 [15]. Nevertheless, there had previously been BIA-DLBCL cases without checking EBV status [22,23] and cases of other EBER+ lymphomas adjacent to breast implants [24]. To our knowledge, only 17 cases of BIA-FA-LBCL had been reported prior to our study (Table 1) [1,10,11,12,13,14,15,16]. Although a case of invasive BIA-FA-LBCL had been reported [16], we will discuss later why we believe that this diagnosis should be taken with caution and why it was not considered a case of BIA-FA-LBCL in this review.

Clinically, all cases have been related to textured implants, and 16 of the 17 patients were immunocompetent (one had a previous history of well-controlled human immunodeficiency virus). Although most of the cases began with pain and capsular contracture, there have also been reports of asymptomatic cases [1,10,11]. The median age at diagnosis was 65 years, with a median of 10 years since the breast implant placement [16]. All cases had been diagnosed at an early stage (IE), and no B symptoms had been reported.

From a histological point of view, large pleomorphic cells are typically distributed in sheets, clusters and ribbons on the luminal side of the capsule. They can also be suspended in a fibrinoid material with thickening and superficial invasion of the capsule, but the malignant cells do not invade pre-existing breast parenchyma. Similar to our case, necrotic material with a sparse inflammatory infiltrate is frequent. These cells expressed B-cell markers such as CD20 (15 of 16 patients tested), PAX-5 (15/17) and CD79a (14/14). They also expressed CD30 (16/17), and they tended to have an activated phenotype following the Hans algorithm (only two cases had a germinal center phenotype). The Ki67 proliferation index was high, with most cases having an 80 percent or higher rate. As the name implies, EBER positivity was always present, commonly with an EBV type III latency pattern [16]. Aberrant expression of T-cell markers and PD-L1 have also been described in single cases [11,13]. Few cases with MYC rearrangements have been reported [11].

The available evidence suggests a good prognosis with detection at an early stage. As in BIA-ALCL, surgical management appears to be sufficient without requiring adjuvant therapy in localized stages. Only three of the 17 cases described received adjuvant chemotherapy, not including the case with invasive disease [16]. To our knowledge, all of them have had favorable outcomes without evidence of relapse to date. Thus, the published data support surgery as the best approach in this situation.

## 5. Discussion

### 5.1. BIA-FA-LBCL in the New WHO and ICC Classification

During the last couple of years, there has been debate about whether these BIA-FA-LBCLs are a new presentation of a known subtype of DLBCL or are a new subtype of their own. In 2022, two new classifications were published: the WHO 2022 [3,4] and the ICC [5].

The ICC does not specifically address this debate, and there are few changes regarding CI-DLBCL or FA-LBCL from the WHO revised 4th edition [2], with the latter being a subtype of the former. Given that this classification has not undertaken the BIA-FA-LBCL debate, we will be focusing on the WHO classification.

The WHO 2022 classification has major changes from its predecessor, and it is clear about how BIA-FA-LBCL should be classified.

The first change related to BIA-FA-LBCL is that, in the WHO 2016 classification, FA-LBCL had been a subtype of CI-DLBCL [2]; in the new classification, however, they have been separated into two different DLBCL subtypes [3,4]. This has been justified because, even though FA-LBCL and CI-DLBCL have in common the inflammatory background, the B-cell phenotype, the morphology of malignant cells and the EBV positivity, they are complete opposites concerning their clinical presentation, management and outcome [3].

Second, the WHO classifies these BIA-FA-LBCLs as FA-LBCL, because they found that the presentation, treatment and prognosis of the BIA-FA-LBCLs and FA-LBCLs developed in other locations are the same [3].

FA-LBCL is described in the novel WHO classification as a neoplasm of large B-cells found incidentally at sites of chronic fibrin deposition in confined natural or acquired anatomic spaces [3]. In the revised 4th edition, FA-LBCL had been described in left atrial myxomas, thrombi/hematomas associated with peripheral vascular disease, chronic hematomas, endovascular prostheses and pseudocysts located within the spleen, adrenal glands or the retroperitoneum [2,25]. Now, the peri-implant space of breast implants is also considered a potential site for FA-LBCL to develop [3]. The symptoms associated with this type of lymphoma differ depending on the localization of the disease [3]. The median age at diagnosis is 59.5 years [3], and even though it has been described as more frequent in males [25], some cases (such as BIA-FA-LBCLs) have a sex bias. In cases with a foreign body involved, it takes a median time of 9 years from implantation for the lymphoma to develop. All cases reported have been localized, incidental microscopic findings [25]. Even though infiltration of myxomatous stroma or fibrous capsular tissue can be found, by definition, infiltration into pre-existing normal parenchymal tissue or mass formation is absent [3]. Due to its indolent nature, most cases have received only surgical intervention, with few cases of persistent disease or relapse [3,25].

The microscopic evaluation shows clusters, ribbons or sheets of large pleomorphic lymphoid cells with prominent nucleoli. These cells appear within superficial layers of fibrin or within a background of fibrin and degenerate cellular debris. In the cases of pseudocysts and chronic hematomas, a similar pattern to BIA-ALCL can be observed, with band-like lymphoplasmacytic infiltrates in the outer layer of the fibrous wall and an inner layer of tumor cells adjacent to fibrin or cyst debris. Inflammation is usually sparse, and even though most cases are EBV+, there have been some rare EBV-negative cases [3]. The malignant cells express B-cell markers (CD20, PAX5 and CD79a) and tend to have a non-germinal center phenotype (CD10 negative, MUM1 positive) [3,24]. They can express CD30, BCL2, BCL6 and PD-L1. The Ki67 is high (>90%), and the EBV+ cases have a EBV type III latency pattern [3,25]. Only rarely have BCL6 and MYC rearrangement been reported [3].

The published literature on all of the non-invasive BIA-FA-LBCLs supports the decision of the WHO to classify them as FA-LBCLs because they match the definition as established in their 5th edition as well as the clinical and histological characteristics found in other FA-LBCLs not related to breast implants.

In relation to the patient with the diagnosis of invasive BIA-FA-LBCL described by Medeiros et al. [16], we believe that the label of BIA-FA-LBCL in this case should be taken with caution due to the fact that she had parenchymal involvement from the beginning (which does not fit de FA-LBCL definition), and the fact that she relapsed at multiple sites and required various lines of chemotherapy for curation (which has not been described in other FA-LBCLs).

### 5.2. Differential Diagnosis: BIA-ALCL and Other EBV+ DLBCLs

#### 5.2.1. BIA-ALCL

The differences between BIA-ALCL and BIA-FA-LBCL have already been analyzed by other authors [16]; however, given that it is still the main differential diagnosis, we will address them briefly. Although BIA-ALCL’s median age of diagnosis is somewhat younger than that of BIA-FA-LBCLs (mid-50s), the median interval from implantation to diagnosis is similar (7 to 10 years), with all cases being related to textured implants [3,4]. BIA-ALCL tends to present as a painful effusion around the implant [6,7]; however, breast masses and axillar lymphadenopathies are also relatively frequent (unlike BIA-FA-LBCLs). Rarely, B symptoms, capsular contractures, cutaneous lesions or even asymptomatic cases have been described [7]. The most common appearance is in an early stage, with an excellent prognosis after surgery alone. Although advanced-stage disease has a poorer prognosis despite the use of chemotherapy, the overall prognosis is still good [3,6].

From the anatomic point of view, most cases present as a seroma-confined disease. BIA-ALCL is defined by a layer of tumor cells caught in a fibrinoid and extensive necrotic meshwork along the luminal side of the capsule [3,7]. Hallmark cells are present in 70% of cases, and malignant cells are large, with pleomorphic and anaplastic morphology. The cells tend to form cohesive clusters embedded in the fibrous exudate adjacent to the implant. In the infiltrating stages, these clusters invade the fibrous capsule and/or the tissue beyond. The lymph node involvement in advanced stages is usually sinusoidal. By definition, the neoplastic cells are strongly positive for CD30. Positivity for CD4 is also typical, as is the expression of cytotoxic markers. The cells lack expression of ALK and other T-cell markers, such as CD3, CD5 and CD7. B-cell markers and EBER are also negative [5]. Clonal rearrangements of the TCR gene can be demonstrated in >80% of cases [3].

Despite their clear differences, from the clinical presentation to the histology, the diagnosis between BIA-ALCL and BIA-FA-LBCL has been proven to be occasionally challenging, as shown by the report of Mansy et al. [14]. Given that BIA-FA-LBCLs have only recently been reported, it is unclear whether the similarities of these two diseases have led to the misclassification of some cases in the past.

#### 5.2.2. EBV+ DLBCL and CI-DLBCL

In addition to the comparison of the characteristics of BIA-FA-LBCLs with BIA-ALCL, we compared them with other EBV+ DLBCLs, to further emphasize their differences. We focused on the most common DLBCLs with EBV positivity in immunocompetent patients: EBV+ DLBCL and CI-DLBCL. The differential diagnosis of BIA-FA-LBCLs is summarized in Table 2.

EBV+ DLBCL (EBV-associated DLBCL of the elderly before the WHO 2016, EBV+ DLBCL-NOS until WHO 2022 and still for the ICC) was first described in 2003 by Oyama et al. [26]. It is a rare entity, representing approximately 5% of all DLBCLs. The diagnosis requires clonal B-cell lymphoid proliferation, with most of the malignant cells expressing EBER in patients with no documented immunodeficiency [2,3,5,6,18]. The median age at diagnosis is 68.5 years, with male predominance. It has an aggressive presentation: advanced-stage disease (III–IV), B symptoms, elevated LDH, Eastern Cooperative Oncology Group-Performance Status ≥ 2 and extranodal disease [17,18]. Younger patients (<45 years) often show nodal involvement, with extranodal manifestations being more frequent in elderly patients [3]. Due to its aggressive nature, treatment with immune-chemotherapy is the main intent-to-cure treatment. EBV DNA is usually detectable in serum or whole blood [3]. Although there is considerable variability between studies in relation to the PFS and OS, to date, EBV+ DLBCL is still considered to have a poorer outcome than EBV− DLBCL-NOS [3,17,19].

Histologically, EBV+ DLBCL can have two different patterns: polymorphic or monomorphic, with no clinical or prognostic implications. Malignant cells are large, with conserved B-cell markers (expressing CD20, PAX-5 and CD79a) and an activated B-cell phenotype following the Hans algorithm. These cells also tend to express CD30 and an EBV type II latency pattern. PD-L1 and PD-L2 are often expressed in younger patients [3]. The polymorphic pattern is characterized by large cells, Hodgkin/Reed–Sternberg-like and lymphocyte-predominant-like cells scattered in a reactive background [3]. The monomorphic type, which is less common, is indistinguishable from EBV-negative DLBCL from a morphological point of view [3]. Rearrangements of MYC, BCL2 and BCL6 are infrequent [17,18,19].

The differences between EBV+ DLBCL and BIA-FA-LBCL are quite evident from a clinical point of view because it is a much more aggressive disease with nodal and extranodal involvement that requires chemotherapy treatment. Histologically, they can exhibit more similarities, given that they both have large pleomorphic cells with an activated B-cell phenotype; however, they do not share either the EBV latency pattern or the distribution patterns of neoplastic cells.

CI-DLBCL is a rare EBV+ neoplasm that occurs in immunocompetent patients with long-standing chronic inflammation involving natural body spaces or acquired tissue spaces [3,17]. Pyothorax-associated lymphoma is the most frequent clinical presentation, representing approximately 80% of all CI-DLBCL [27]. It was first described in 1987 by Aozasa et al. [21], and it has been associated with artificial pneumothorax, chronic osteomyelitis, metallic implants and chronic skin ulcers [2,3,4,5]. The median age at diagnosis is 67 years, with a median of 37–43 years since the onset of the pyothorax, and it has a clear male predominance. Clinically, it is very aggressive, with tumor masses that involve the pleura and directly invade adjacent structures. Pain and constitutional symptoms without node or bone marrow involvement are common [3,20]. In cases not related to pyothorax, the interval from the predisposing event to the development of the lymphoma is more than 10 years [3]. CI-DLBCL has an ominous prognosis, with OS at 5 years of 22% despite treatment with systemic chemotherapy [3,17].

Histologically, the affected tissues show a background with marked fibrous thickening with relatively sparse nonneoplastic inflammatory cells. The malignant cells are large with a diffuse pattern and large nucleoli. They can have centroblastic or immunoblastic features, and massive necrosis and angiocentric growth can be present [3]. Although cells tend to express B-cell markers with an activated phenotype, they can also exhibit plasmacytoid differentiation and show positivity for CD138 and MUM1. They normally have a type III EBV latency pattern and a high Ki67. Occasionally, they can express CD30 and aberrant T-cell markers such as CD3 and CD4 [3]. MYC amplifications, TP53 mutations and complex karyotypes are frequent [17,26,28].

Differentiating between CI-DLBCL and BIA-FA-LBCL can be more challenging from a histological point of view, given that they share B-cell markers, the EBV latency pattern and the activated B-cell phenotype. In fact, the distinction between the two is not possible in small specimens such as core needle biopsies [3]. However, employing an integrated approach to the patient, the aggressiveness of CI-DLBCL and the fact that they are prone to have tumor masses can facilitate the differentiation between both entities.

## 6. Conclusions

We believe our findings reaffirm the decision of the 5th edition of the WHO classification of Tumors of Hematopoietic and Lymphoid Tissues to identify the BIA-FA-LBCLs as FA-LBCL. Previously described FA-LBCLs share the same histological characteristics, indolent behavior and excellent prognosis with surgical management as those we found in the BIA-FA-LBCL reports. We also believe this literature review highlights the main challenges in the diagnosis of this new face of FA-LBCL. The absence of tumor masses or parenchymal involvement should be the first step to differentiate these lymphomas from other EBV+ DLBCLs or infiltrating BIA-ALCLs. In cases affecting only the implant capsule, T-cell markers, B-cell markers and EBER testing should be performed to help differentiate FA-LBCL arising in the context of breast implants from BIA-ALCL. We hope our case and this literature review helps raise awareness of this new FA-LBCL presentation, adding to the literature published thus far.

## Figures and Tables

**Table 1:** BIA-FA-LBCLs previously described in literature, including the current case. N/A: data not available; ND: not done; DLBCL: diffuse large B-cell lymphoma; EBER: Epstein–Barr virus-encoded small RNA; CI-DLBCL: diffuse large B-cell lymphoma associated with chronic inflammation; FA-LBCL: fibrin-associated large B-cell lymphoma; HIV: human immunodeficiency virus; R-CHOP: Rituximab, Cyclophosphamide, Doxorubicin, Vincristine, Prednisone; BEACOPP: Bleomycin, Etoposide, Adriamycin, Cyclophosphamide, Vincristine, Procarbazine, Prednisone; RT: radiation therapy; ICE: Ifosfamide, Carboplatin, Etoposide; ASCT: autologous stem cell transplant. * Latency time: time from implant to lymphoma (years). ^†^ The outcome was unknown because the patient was lost to follow-up. ^‡^ Two of the 7 non-invasive cases reported by Medeiros et al., 2021 [16], have been previously published by Mescam et al., 2020 [11], and Khoo et al., 2020 [12], respectively.

	PATIENT FEATURES	IMPLANTS	CLINICAL PRESENTATION	LATENCY TIME *	LYMPHOMA HYSTOLOGY	DISEASE DISTRIBUTION	TREATMENT STRATEGY	OUTCOME
Goodwins et al., 2019 [15]	66-years female	Textured silicone	Bilateral capsular contracture	12 years	DLBCL, EBER+ (latency pattern ND)	Early-stage (IE)	Surgery (complete capsulectomy)	Complete remission
Rodríguez Pinilla et al., 2020 [10]	55-years female	N/A	Hematoma	15 years	DLBCL, EBER+ (type III latency)	Early-stage (IE)	Surgery (complete capsulectomy)	Complete remission
Rodríguez Pinilla et al., 2020 [10]	59-years female	N/A	Hematoma	10 years	DLBCL, EBER+ (type III latency)	Early-stage (IE)	Surgery (complete capsulectomy)	Complete remission
Rodríguez Pinilla et al., 2020 [10]	63-years female	N/A	Tumor mass	20 years	DLBCL, EBER+ (type III latency)	Early-stage (IE)	Surgery (complete capsulectomy)	N/A ^†^
Mescam et al., 2020 [11]	72-years female	Textured silicone	No symptoms	8 years	CI-DLBCL, EBER+ (type III latency)	Early-stage (IE)	Surgery (complete capsulectomy)	Complete remission
Mescam et al., 2020 [11]	61-years female	Textured silicone	No symptoms	13 years	CI-DLBCL, EBER+ (type I latency)	Early-stage (IE)	Surgery (complete capsulectomy)	Complete remission
Mescam et al., 2020 [11]	69-years female	Textured silicone	No symptoms	9 years	CI-DLBCL, EBER+ (type III latency)	Early-stage (IE)	Surgery (complete capsulectomy) + Chemotherapy (3 R-CHOP cycles)	Complete remission
Khoo et al., 2020 [12]	70-years female	Textured silicone	Right capsular contracture	9 years	FA-LBCL, EBER+ (latency pattern ND)	Early-stage (IE)	Surgery (complete capsulectomy)	Complete remission
Malata et al., 2021 [13]	51-years female, HIV well controlled	Textured silicone (previous smooth- saline)	Left capsular contracture	15 years (21 years from saline implants)	DLBCL, EBER+ (latency pattern ND)	Early-stage (IE)	None	Complete remission
Medeiros et al., 2021 ^‡^ (7 cases) [16]	Median: 65-years females (range: 47–71)	Textured silicone (4), saline (1), N/A (2)	Capsular contracture (6), no symptoms (1)	Median: 10 years (range: 4–26)	DLBCL, EBER+ (type III latency)	Early-stage (IE)	Surgery (complete capsulectomy) +/− Chemotherapy (3 R-CHOP) (1)	Complete remission
Medeiros et al., 2021 (1 case) [16]	48-years female	Silicone (surface N/A)	Invasive mass (left breast parenchyma)	21 years	DLBCL, EBER+ (type II latency)	Early-stage (IE)	Surgery (complete capsulectomy) + Chemotherapy (4 R-CHOP, 2 BEACOPP + RT, 1 ICE + ASCT)	Complete remission after 2 relapses
Morgan et al., 2021 [1]	69-years female	Textured silicone	Right capsular contracture	12 years	DLBCL, EBER+ (latency pattern ND)	Early-stage (IE)	Surgery (complete capsulectomy)	Complete remission
Morgan et al., 2021 [1]	53-years female	Textured silicone	Bilateral capsular contracture	9 years	DLBCL, EBER+ (latency pattern ND)	Early-stage (IE)	Surgery (complete capsulectomy) + Chemotherapy (4 R-CHOP)	Complete remission
Mansy et al., 2021 [14]	46-years female	Textured silicone	Bilateral capsular contracture	8 years	CI-DLBCL, EBER+ (type III latency)	Early-stage (IE)	Surgery (complete capsulectomy)	Complete remission
Current case	42-years female	Textured silicone	Bilateral capsular contracture	7 years	DLBCL, EBER+ (latency pattern ND)	Early-stage (IE)	Surgery (complete capsulectomy)	Complete remission

**Table 2:** Summary of BIA-FA-LBCL, BIA-ALCL, FA-LBCL, CI-DLBCL and EBV+ DLBCL characteristics. BIA-FA-LBCL: breast implant-associated fibrin-associated large B-cell lymphoma; BIA-ALCL: breast implant-associated anaplastic large cell lymphoma; FA-LBCL: fibrin-associated large B-cell lymphoma; CI-DLBCL: diffuse large B-cell lymphoma associated with chronic inflammation; EBV+ DLBCL: Epstein–Barr virus-positive diffuse large B-Cell lymphoma; EBV: Epstein–Barr virus; N/A: non-applicable; IHC: immunohistochemistry.

	BIA-FA-LBCL	BIA-ALCL	FA-LBCL	CI-DLBCL	EBV + DLBCL
Clinical Features	Capsular contracture (indolent)	Capsular contracture (indolent)	Incidental (indolent)	Pyothorax, inflammation (aggressive)	B symptoms (aggressive)
Disease distribution	Capsule localized	Capsule localized	Defined anatomic spaces (myxomas, vascular prostheses)	Pleural, metallic implant	Nodal and extranodal
Stage of disease at diagnosis	I	I	I	I/II	III/IV
Association with EBV	+++	−	+++	+	+++
EBV latency pattern	III	−	III	III	II > III
Onset inflammatory stimulus	10 years	7–10 years	9 years	37–43 years	N/A
Histologic features	Pleomorphic cells (non-germinal center), Fibrin deposition	Large and pleomorphic cells	Pleomorphic cells (non-germinal center), fibrin deposition +/− inflammatory infiltrate	Plasmablastic/immunoblastic (non-germinal center), inflammatory infiltrate	Polymorphic cells (non-germinal center)
IHC markers	CD20, CD30+, CD79a, PAX-5	CD30, CD4+/−	CD20, CD30+, CD79a+, PAX-5, PD-L1+	CD20, CD30 +/, CD4	CD20, CD30+/−, CD79a, PAX-5, PD-L1+
Ki67	High	High	>90%	>90%	>90%
Molecular analysis	Few data, few cases MYC rearrangements	ALK-	Rarely MYC rearrangements	TP53 mutations	Infrequent MYC, BCL-2, BCL-6 rearrangements

## Data Availability

No new data were created in this study. Data sharing is not applicable to this article.
